# High Caloric Diet Induces Memory Impairment and Disrupts Synaptic Plasticity in Aged Rats

**DOI:** 10.3390/cimb43030162

**Published:** 2021-12-18

**Authors:** Sara L. Paulo, Catarina Miranda-Lourenço, Rita F. Belo, Rui S. Rodrigues, João Fonseca-Gomes, Sara R. Tanqueiro, Vera Geraldes, Isabel Rocha, Ana M. Sebastião, Sara Xapelli, Maria J. Diógenes

**Affiliations:** 1 Instituto de Farmacologia e Neurociências, Faculdade de Medicina, Universidade de Lisboa, 1649-028 Lisboa, Portugal; sara.paulo@medicina.ulisboa.pt (S.L.P.); catarinalourenco@medicina.ulisboa.pt (C.M.-L.); rbelo@medicina.ulisboa.pt (R.F.B.); rmsrodrigues@medicina.ulisboa.pt (R.S.R.); joaogomes@medicina.ulisboa.pt (J.F.-G.); saratanqueiro@hotmail.com (S.R.T.); anaseb@medicina.ulisboa.pt (A.M.S.); sxapelli@medicina.ulisboa.pt (S.X.); 2 Instituto de Medicina Molecular João Lobo Antunes, Faculdade de Medicina, Universidade de Lisboa, 1649-028 Lisboa, Portugal; 3 Instituto de Fisiologia, Faculdade de Medicina, Universidade de Lisboa, 1649-028 Lisboa, Portugal; vgeraldes@medicina.ulisboa.pt (V.G.); isabelrocha@gmail.com (I.R.); 4 Centro Cardiovascular da Universidade de Lisboa, 1649-028 Lisboa, Portugal

**Keywords:** aging, high caloric diet, obesity, memory, hippocampal plasticity, brain-derived neurotrophic factor, neurogenesis

## Abstract

The increasing consumption of sugar and fat seen over the last decades and the consequent overweight and obesity, were recently linked with a deleterious effect on cognition and synaptic function. A major question, which remains to be clarified, is whether obesity in the elderly is an additional risk factor for cognitive impairment. We aimed at unravelling the impact of a chronic high caloric diet (HCD) on memory performance and synaptic plasticity in aged rats. Male rats were kept on an HCD or a standard diet (control) from 1 to 24 months of age. The results showed that under an HCD, aged rats were obese and displayed significant long-term recognition memory impairment when compared to age-matched controls. Ex vivo synaptic plasticity recorded from hippocampal slices from HCD-fed aged rats revealed a reduction in the magnitude of long-term potentiation, accompanied by a decrease in the levels of the brain-derived neurotrophic factor receptors TrkB full-length (TrkB-FL). No alterations in neurogenesis were observed, as quantified by the density of immature doublecortin-positive neurons in the hippocampal dentate gyrus. This study highlights that obesity induced by a chronic HCD exacerbates age-associated cognitive decline, likely due to impaired synaptic plasticity, which might be associated with deficits in TrkB-FL signaling.

## 1. Introduction

Diet plays a vast role in global health, and the increasing consumption of sugar and fat, seen over the last decades, is leading to the growing prevalence of obesity in all age groups [1,2]. Moreover, contemporary high caloric diets (HCDs), highly prevalent in the western world, are characterized by high levels of saturated fat and refined sugars [1], which might be contributing to the worsening of this scenario [3]. In fact, obesity has emerged as one of the major health problems in the modern world [4], and it has been related with multiple comorbidities, including cancer, metabolic and cardiovascular problems, as well as cognitive decline [1,5,6,7,8,9,10]. With the increase in average life expectancy, providing conditions to maintain elderly cognitive function is of great concern. However, whether HCD-induced obesity impacts brain function during aging remains poorly understood. Nonetheless, evidence from adult rodents suggests that the hippocampus, a cerebral area crucial for learning and memory, might be strongly affected by HCDs [11]. Accordingly, three strong players have been associated with this hippocampal dysfunction: increased neuroinflammation, weakened brain-blood barrier integrity, and downregulation of brain-derived neurotrophic factor (BDNF) [12]. BDNF is a neurotrophic factor highly relevant for the regulation of neuronal survival, differentiation and maturation, synaptic plasticity and long-term memory [13]. Importantly, BDNF signaling, through its high-affinity receptor, tropomyosin receptor kinase B-full length (TrkB-FL), has been shown to enhance hippocampal-related plasticity mechanisms such as long-term potentiation (LTP), a crucial mechanism for learning and memory processes, and adult neurogenesis [14,15], while impaired signaling has been associated with cognitive decline in old age [16].

In the elderly, overweight represents an additional risk factor for individuals with other chronic disorders [1]. Recent studies have shown that obesity and its associated risk factors are linked to a higher risk of developing mild cognitive impairment, late-onset Alzheimer’s disease and dementia [17,18,19]. In adult mice, HCD consumption was shown to have a negative impact upon memory [20]. Similarly, deficits in tasks requiring hippocampal and cortical function were shown in adult rodents fed HCDs [12,21]. In addition, neurogenesis in the hippocampal dentate gyrus (DG) constitutes an important mechanism of brain plasticity, also contributing to learning and memory [22]. This process is highly regulated by nutritional components, with studies showing downregulated adult hippocampal neurogenesis after exposing young rodents to high-fat and/or high-sugar diets [23]. However, during aging, this effect remains to be clarified.

The evidence available so far shows that HCD consumption is a risk factor for cognitive impairment in adults. It is also known that BDNF levels are altered in the hippocampus of adult rodents fed HCDs [24,25]. Thus, we hypothesized that a chronic HCD contributes to the cognitive decline in aged rats and that this could be related to altered synaptic plasticity and BDNF signaling. We directly addressed this hypothesis by assessing long-term episodic memory, hippocampal LTP, TrkB-FL levels and neurogenesis in aged rats fed an HCD throughout life, in comparison with age-matched rats fed a control (CTL) standard chow diet.

## 2. Materials and Methods

### 2.1. Animals

**Figure 1:**
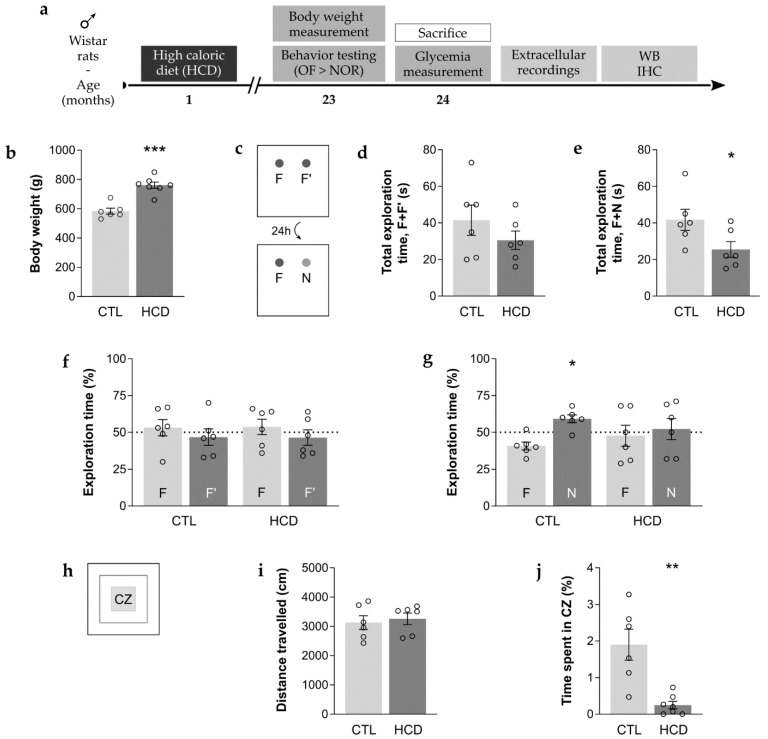
A chronic high caloric diet (HCD) induced obesity, enhanced long-term memory impairment and anxious-related behavior. (**a**) Experimental timeline. Rats were kept on a high caloric diet (HCD) starting at 1 month of age, until their sacrifice at 24 months of age. Behavior assessment was performed in the open field (OF) test, followed by the novel object recognition (NOR) test at 23 months of age. Body weight was measured immediately prior to behavior testing. Post-mortem tissue samples were analyzed by electrophysiological extracellular recordings, western blot (WB) and immunohistochemistry (IHC). (**b**) Significant increase in body weight, leading to obesity, was observed in 23-month-old HCD rats. *** *p* < 0.001, unpaired Student’s *t*-test. (**c**–**g**) Long-term episodic memory of 23-month-old HCD rats was impaired in the NOR test. (**d**,**f**) Performance in the training phase. (**e**,**g**) Performance in the test phase. (**d**) No significant changes in the total exploration time of the two familiar objects (F, F’). *p* > 0.05, unpaired Student’s *t*-test. (**e**) Significant decrease in the total exploration time of the familiar (F) plus the novel object (N). * *p* < 0.05, unpaired Student’s *t*-test. (**f**) Both CTL and HCD aged rats showed no preference for any of the familiar objects (F, F’). *p* > 0.05, paired Student’s *t*-test. (**g**) CTL aged rats were able to distinguish the novel (N) from the familiar (F) object, while HCD aged rats were not. * *p* < 0.05, *p* > 0.05, paired Student’s *t*-test. (**g**–**j**) Anxious-related behavior was enhanced, despite preserved locomotor activity of 23-month-old HCD rats in the OF test. (**i**) Locomotor activity. (**j**) Anxious-related behavior. (**i**) No significant changes in distance travelled, * *p* > 0.05, Mann–Whitney test. (**j**) Significant decrease in the time spent in the central zone (CZ) of the apparatus. ** *p* < 0.01, unpaired Student’s *t*-test. (**b**–**j**) Data are expressed as means ± SEM.

Male Wistar rats (Charles River, Barcelona, Spain), after being weaned for 30–37 days, were randomly divided into two groups, depending on the diet followed. Rats were housed under regulated temperature and light conditions (12 h dark/light cycle). All experimental procedures were conducted in conformity with the European Community legislation (86/609/EEC; Directive 2010/63/EU, 2012/707/EU) and approved by the Ethical Committee of the Faculty of Medicine, University of Lisbon, as well as by the Direção Geral de Alimentação e Veterinária (DGAV), the Portuguese competent authority for animal protection. All efforts were made to minimize animal suffering to the greatest extent. Handling of the rats was performed for 3 days before any procedure. The rats were sacrificed at 24 months of age (Figure 1a).

#### Diet

A combination of sucrose solution (33%) and a solid mixture of lard (33%) and powdered sucrose (66%) was used as the source of a chronic HCD, given from 1 to 24 months of age (Figure 1a). Water and standard rat chow (19% protein, 3% fat and 61% carbohydrates) (4RF21, Mucedola, Milan, Italy) were also available. Food and drinking solutions were available ad libitum for the rats to choose the type and amount of diet ingested. Age-matched CTL rats were fed standard rat chow daily in the controlled amount of 24 g, corresponding to an average calorie ingestion. Body weight was measured immediately prior to behavior assessment (Figure 1a).

### 2.2. Behavior Assessment

All behavior tests were performed when the rats reached 23 months of age (Figure 1a), during the light phase of the light/dark cycle, under dim light conditions. Olfactory clues were erased from the corresponding objects and/or apparatus between each trial.

#### 2.2.1. Open Field (OF) Test

The OF test grants the opportunity to simultaneously appraise rodent locomotor activity and anxious-related behavior [26,27]. Each rat was individually placed in the center of an empty square box (60 cm × 60 cm × 40 cm) and allowed to move freely for 5 min without prior habituation. The area was virtually divided into three concentric squares at a similar distance from each other, defining a peripheral, an intermediate and a central zone (CZ). Parameters were recorded using the SMART V2.5 video-tracking software (Barcelona, Spain). Total distance travelled (cm) was used to assess locomotion and exploratory drive, while time spent in the CZ (%) was considered an inverse measure of anxiety-like behavior.

#### 2.2.2. Novel Object Recognition (NOR) Test

The NOR test is particularly useful for evaluation of memory performance in rodents, namely episodic long-term memory, which is considered to be related to hippocampal function [27,28]. In the habituation phase (3 consecutive days), each rat freely explored the empty OF test arena for 15 min. The first 5 min of the first habituation day corresponded to the OF test. During the familiarization phase (in the fourth day), rats were presented with two identical objects (the familiar objects, F + F’) for 5 min. The test phase was conducted after a 24 h intertrial interval, where one familiar object was replaced by a novel object (N), and the rats returned to the arena for 5 min. The used objects consisted of two brown and two transparent glass bottles (5 cm in base diameter, 22 cm in height) filled with water, used interchangeably as familiar and novel objects.

The test was video recorded and posteriorly analyzed by two independent observers blind to the experimental conditions. The time (s) that the rat spent exploring each of the objects was manually recorded. Exploration was scored whenever the rat touched an object with its forepaws and/or bit, sniffed or reared towards the object. Total exploration time (s) of both objects in the familiarization or test phase was used as a measure of exploratory drive. Concerning long-term memory performance, the results are presented as the exploration time (%) for each object and for each phase being the time spent exploring that object divided by the total time spent exploring both objects.

### 2.3. Electrophysiological Recordings

#### 2.3.1. Acute Hippocampal Slices

The rats were sacrificed at 24 months of age (Figure 1a) by decapitation, following deep anesthesia with isoflurane (1,2-propylene glycol 50% (*v*/*v*)) (Esteve, Barcelona, Spain). The brains were quickly removed and both hippocampi were dissected in previously oxygenated (O_2_/CO_2_: 95/5%) ice-cold artificial cerebrospinal fluid (aCSF) (124 mM NaCl, 3 mM KCl, 1.25 mM NaH_2_PO_4_, 26 mM NaHCO_3_, 1 mM MgSO_4_, 2 mM CaCl_2_ and 10 mM glucose, pH 7.4). Then, each hippocampus was cut perpendicularly to the long axis into 400 μm thick slices with the McIlwain tissue chopper (The Mickle Laboratory Engineering Co. Ltd., Surrey, UK). The slices were placed in a resting chamber with an aCSF solution that was continuously oxygenated at room temperature (RT) for at least 1 h in order to recover.

#### 2.3.2. Field Excitatory Postsynaptic Potentials (fEPSPs) Quantification and LTP Induction

**Figure 2:**
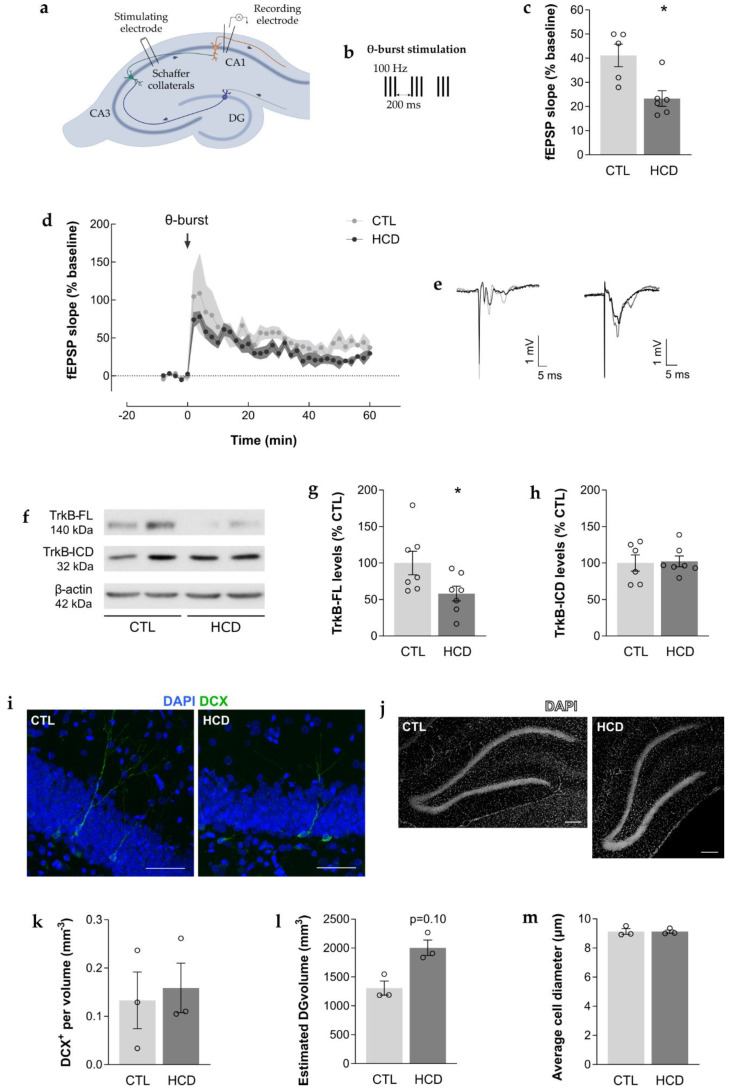
A chronic high caloric diet (HCD) promoted hippocampal synaptic plasticity impairment and reduced the levels of hippocampal TrkB-FL without impacting neurogenesis. (**a**,**b**) Representative scheme of electrophysiological recordings in acute hippocampal slices. (**a**) Schematic representation of an acute hippocampal slice with the electrophysiological recording configuration used to obtain field excitatory postsynaptic potentials (fEPSPs) from the CA1 area under the stimulation of Schaffer collateral/commissural fibers in the stratum radiatum of the CA1 area. (**b**) Representation of the applied long-term potentiation (LTP) protocol. After a stable baseline (10 min), LTP was induced through a weak θ-burst protocol (3 trains of 100 Hz, 3 stimuli, separated by 200 ms). (**c**–**e**) Hippocampal synaptic plasticity of 24-month-old HCD rats was impaired, as assessed by electrophysiological extracellular recordings. (**c**) Significant decrease in LTP magnitude, quantified as the fEPSP average slope (% baseline) obtained between 50 and 60 min after LTP induction (θ-burst). * *p* < 0.05, unpaired Student’s *t*-test. Data are expressed as mean % ± SEM. (**d**) The averaged time-course changes in fEPSP slope (% baseline) induced by a θ-burst stimulation. Data are expressed as mean % ± SEM. (**e**) Tracings from representative experiments. For each condition, fEPSP tracings recorded at baseline (baseline, grey line) and after θ-burst-induced LTP (LTP, black line) from the same slice are shown overlaid. (**f**–**h**) Levels of TrkB full length (TrkB-FL) were reduced in the hippocampus of 24 month old HCD rats, as assessed by western blot (WB). (**f**) Representative WBs depict immunoreactive bands for TrkB-FL (~140 kDa), TrkB-ICD (~32 kDa) and β-actin (loading control, ~42 kDa). (**g**,**h**) Protein levels were quantified and normalized (100%) for the corresponding controls (% CTL). (**g**) Significant decrease in the levels of TrkB-FL, * *p* < 0.05, unpaired Student’s *t*-test, which did not result in (**h**) changes in the levels of TrkB intracellular domain fragment (TrkB-ICD), *p* > 0.05, unpaired Student’s *t*-test. (**i**–**m**) The density of immature neurons was not altered in the dentate gyrus (DG) of the hippocampus of 24-month-old HCD rats, as assessed by IHC. (**i**) Representative coronal sections immunostained with 4′,6-diamidino-2-phenylindole (DAPI) (blue) and doublecortin (DCX) (green). Scale bar = 50 μm. (**j**) Representative coronal sections immunostained with DAPI (white). Scale bar = 200 μm. (**k**) No significant difference in the density of DCX+ cells. * *p* > 0.05, Mann–Whitney test. (**l**) No significant differences in the estimated volume of the DG or (**m**) in the average diameter of DAPI+ cells. *p* > 0.05, unpaired Student’s t-test. (**c**,**d**) Data are expressed as mean % ± SEM. (**g**,**h**,**k**–**m**) Data are expressed as mean ± SEM.

The hippocampal slices were transferred to a recording chamber that was continuously superfused with oxygenated aCSF at 32 °C (3 mL/min flow rate). Stimulation was performed using two bipolar concentric wire electrodes placed on Schaffer collateral/commissural fibers in the stratum radiatum of the CA1 area, allowing alternated stimulation of two independent pathways (rectangular 0.1 ms pulses, delivered every 10 s). fEPSPs were recorded using an extracellular microelectrode filled with aCSF (2–8 MΩ resistance, Harvard apparatus Ltd., MA, USA) placed in the stratum radiatum of the CA1 area (Figure 2a), and recordings were obtained with an Axoclamp 2B amplifier (Axon Instruments, Foster City, CA, USA), digitized and continuously stored on a personal computer with the WinLTP software [29]. Individual responses were monitored as the averages of six consecutive signals. The slope of the initial phase of the fEPSPs was quantified. After fEPSP stabilization, LTP was induced through a θ-burst protocol (3 trains of 100 Hz, 3 stimuli, with a 200 ms interval) (Figure 2b) in one of the stimulation pathways. The θ-burst-induced LTP pattern of stimulation is considered close to what physiologically occurs in hippocampi during episodes of learning and memory in living rodents [30]. LTP magnitude was quantified as the change (%) in the average slope of fEPSPs during the period of 50 to 60 min after LTP induction compared with the average slope of fEPSPs measured during the 10 min prior to θ-burst-induced LTP (baseline).

### 2.4. Western blot

#### 2.4.1. Protein Extraction

Hippocampal samples were obtained at 24 months of age (Figure 1a), as described for freshly prepared acute hippocampal slices. Dissected tissue was rinsed in phosphate-buffered saline (PBS) (137 mM NaCl, 2.7 mM KCl, 1.8 mM KH_2_PO_4_ and 10 mM Na_2_HPO_4_·2 H_2_O, pH 7.4), snap-frozen in liquid nitrogen and stored at −80 °C. For the analysis, samples were disrupted by sonication in radio-immunoprecipitation assay (RIPA) lysis buffer (50 mM Tris-base pH 7.5, 150 mM NaCl and 1 mM ethylenediamine tetra-acetic acid (EDTA), 0.1% SDS, 4% NP-40 and protease inhibitor cocktail (Mini-Complete EDTA-free, Roche Applied Science, Penzberg, Germany). The lysates were then vortexed, sonicated (3 cycles of 15 s) and clarified with a centrifugation of 16,000× *g* at 4 °C for 10 min. Protein quantification was determined by Bradford assay with Bio-Rad DC reagent (Sigma-Aldrich, St. Louis, MO, USA). All samples were prepared with the same amount of total protein (70 µg), with the addition of a loading buffer (47% glycerol, 12% SDS, 0.06% bromophenol blue, 600 mM dithiothreitol and 60 mM Tris-HCl, pH 6.8) and then denatured at 95 °C for 5 min.

#### 2.4.2. Protein Electrophoresis, Transfer and Detection/Quantification

Samples and molecular weight marker (NZYColour Protein Marker II MB090, NZYTech, Lisbon, Portugal) were loaded and separated on 12% sodium dodecyl sulphate-polyvinylidene gel electrophoresis (SDS-PAGE), before being transferred to methanol-activated polyvinylidene fluoride (PVDF) membranes (GE Healthcare, Chicago, IL, USA). The PVDF membranes were blocked with 3% bovine serum albumin (BSA, NZYTech, Lisbon, Portugal) in Tris-buffered saline with Tween (TBS-T) (20 mM Tris base, 137 mM NaCl and 0.1% Tween^®^ 20, pH 7.6) at RT for 1 h and washed with TBS-T. The membranes were incubated with anti-TrkB, anti-β-Actin and anti-glyceraldehyde-3-phosphate dehydrogenase (GAPDH) primary antibodies (Table 1) overnight at 4 °C, followed by the corresponding HRP-conjugated secondary antibodies (Table 1) for 1 h at RT, all diluted in 3% BSA solution in TBS-T. Immunoreactivity was detected using the ECL chemiluminescence detection system (Amersham-ECL Western Blotting Detection Reagents from GE Healthcare, Chicago, IL, USA) on the ChemiDoc^TM^ XRS^+^ imaging system (Bio-Rad, Hercules, CA, USA) with Image Lab software 5.2.1. Image-J 1.45 software (Bethesda Softworks, Bethesda, MD, USA) was used for the band densitometry analysis, which was normalized to the loading control (see Supplementary Materials). Both β-Actin (*n* = 4) and GAPDH (*n* = 3) were used as loading controls.

### 2.5. Immunohistochemistry

#### 2.5.1. Tissue Processing

After reaching a deep anesthesia state with isoflurane, 24-month-old rats were transcardially perfused with PBS, followed by 4% paraformaldehyde (PFA, VWR, Vila Nova de Gaia, Portugal) in PBS (pH 6.8–7.4) (Figure 1a) [31]. The brains were removed, post-fixed in 4% PFA at 4 °C for 24 h and then submerged with 15% and 30% sucrose in PBS (4 °C). Subsequently, brains were gelatin-embedded and coronally sectioned at 40 µm (cryostat Leica CM3050 S, Leica Biosystems, Wetzlar, Germany). Coronal sections of the hippocampus from the right hemisphere were collected, in ten series, each one comprising an anterior–posterior reconstruction of the whole hippocampus, where sections were separated by 400 µm. Sections were collected to 0.1% sodium azide in PBS in 24-well plates and stored at 4 °C.

#### 2.5.2. Antigen Detection

Free-floating immunohistochemistry (IHC) was performed to assess the presence of immature cells in the DG of the hippocampus. A complete series of slices from each animal, using a specific antibody combination, was considered *n* = 1. Slices were degelatinized in PBS (37 °C) and blocked in a blocking solution (3% BSA and 0.2% Triton^TM^ X-100 in PBS) at RT for 1 h. Slices were incubated overnight with the primary antibody (Table 1) for doublecortin (DCX) (expressed by neuroblasts and immature neurons [32]), prepared in the same blocking solution, at 4 °C. Then, slices were washed in PBS (3 × 10 min) and incubated with the secondary antibody (Table 1) and DAPI (1:1000) (Sigma-Aldrich, St. Louis, MO, USA) in PBS at RT for 2 h. After washing in PBS (3 × 10 min), slices were mounted on microscope slides (Superfrost^TM^ Plus, Thermo Fisher Scientific, Waltham, MA, USA) with Mowiol fluorescent medium and glass coverslips on top.

Images of the DG were captured using a Zeiss LSM 880 with Airyscan (Carl Zeiss, Oberkochen, Germany) confocal point-scanning microscope, with a 20× objective, throughout the entire thickness of each coronal section (40 µm). All analyses were performed by an observer blind to the experimental conditions. The area (mm^2^) of the DG of each section was measured using the ZEN 2.3 software (Carl Zeiss, Oberkochen, Germany). The total DG volume (mm^3^) for each animal was estimated by multiplying the sum of the areas by the distance between representative coronal slices (400 µm). Cell diameter (µm) was also measured using the ZEN 2.3 software for an average of ninety 4′,6-diamidino-2-phenylindole-immuno-positive (DAPI^+^) cells per animal. Quantification of DCX^+^ cells was achieved by manually counting the total number of these cells within the DG layer. Results are presented as the number of DCX^+^ cells per volume (mm^−3^) of DG of each section.

### 2.6. Statistical Analysis

Gathered data is presented as the means ± standard errors of the mean (SEM). All statistical analyses were performed using the software Graphpad Prism 7 (CA, USA) for Windows. Data normality was confirmed using the Shapiro–Wilk test. The obtained results were analyzed using unpaired two-tailed Student’s *t*-tests or Mann–Whitney tests. To assess the significance between differences within the same group, paired two-tailed Student’s *t*-tests were used. Significant outliers were excluded whenever values were more than 2 standard deviations (SD) away from the mean, for Gaussian distributions, or whenever values were more than 1.5 interquartile ranges (IQR) away from the first or third quadrant, for non-Gaussian distributions [33,34]. Differences were considered statistically significant at a *p*-value of < 0.05.

## 3. Results

### 3.1. An Obesogenic HCD Promotes Long-Term Memory Impairment and Enhances Anxious-Related Behavior

Exposure to an HCD for 23 months (Figure 1a) resulted in a significant increase in body weight, leading to obesity when compared with age-matched CTL-fed rats (CTL: 584 ± 20.3 g, HCD: 761 ± 21.5 g, *p* < 0.001, *n* = 6–7, Figure 1b). The influence of the HCD on episodic long-term memory was assessed by the NOR test (Figure 1c) and, as can be concluded from the data shown in Figure 1d–g, HCD rats performed worse than CTL rats during the test phase. The total exploration time of the two objects served as a measure of exploratory drive in both the training and testing phases of this test. No significant differences were observed in this parameter during training between both animal groups (CTL: 41.5 ± 8.29 s, HCD: 30.5 ± 5.04 s, *p* ≥ 0.05, *n* = 6, Figure 1d), although a decrease in exploratory drive was denoted during the test phase in the HCD group (CTL: 41.7 ± 5.77 s, HCD: 25.5 ± 4.31 s, *p* < 0.05, *n* = 6, Figure 1e). In addition, during training, animals showed no preference for either of the identical objects (F and F’), as seen by the absence of differences in the time spent exploring these objects within the CTL group (F: 53.2 ± 5.51%, F’: 46.8 ± 5.51%, *p* ≥ 0.05, *n* = 6, Figure 1f), and within the HCD group (F: 53.7 ± 5.23%, F’: 46.5 ± 5.18%, *p* ≥ 0.05, *n* = 6, Figure 1f). During the test phase, CTL aged rats spent a significantly greater amount of time exploring the novel object (N) when compared to the familiar one (F) (F: 40.8 ± 2.66 %, N: 59.2 ± 2.66%, *p* < 0.05, *n* = 6, Figure 1g), while HCD aged rats spent a similar amount of time exploring both objects (F: 47.7 ± 7.11%, N: 52.3 ± 7.11%, *p* ≥ 0.05, *n* = 6, Figure 1g), reflecting their inability to recognize the novel object.

The OF test (Figure 1h) allowed for the evaluation of the potential impact of the HCD, and subsequent overweight, on locomotor activity, since the latter can affect exploratory drive during the NOR test. There were no significant differences between the CTL and HCD aged rats concerning total distance travelled (CTL: 3127 ± 234.8 cm, HCD: 3259 ± 200.3 cm, *p* ≥ 0.05, *n* = 6, Figure 1i). Moreover, changes in anxiety-related behavior were appraised by quantifying the time that the rats spent in the center of the OF arena. This parameter was shown to be significantly decreased for HCD aged rats (CTL: 1.90 ± 0.424 s, HCD: 0.249 ± 0.102 s, *p* < 0.01, *n* = 6–7, Figure 1j), indicating enhanced anxious-like behavior in these animals.

### 3.2. HCD Induces Hippocampal Synaptic Plasticity Impairment along with Reduced Levels of Hippocampal TrkB-FL

Following the hippocampal-dependent memory impairment observed in the NOR test, synaptic plasticity was evaluated by studying hippocampal θ-burst-induced LTP (Figure 2a,b). The LTP magnitude recorded from hippocampal slices of HCD aged rats was found to be significantly decreased when compared to CTL aged rats (CTL: 41.2 ± 4.68 %, HCD: 23.3 ± 3.21 %, *p* < 0.05, *n* = 5–6, 2–3 slices per animal, Figure 2c–e), highlighting a compromise in hippocampal synaptic plasticity.

To unveil the possible mechanism underlying the synaptic plasticity deficits observed in HCD aged rats, the protein levels of TrkB-FL were assessed. The results showed that TrkB-FL levels were significantly decreased in the hippocampus of HCD aged rats when compared to the CTL group (CTL: 100.0 ± 16.06 %*,* HCD: 58.14 ± 10.15 %, *p* < 0.05, *n* = 7; Figure 2f,g). To understand whether this decrease could be due to the cleavage of TrkB-FL, as seen in excitotoxic conditions [35], leading to the concomitant increase of the levels of its intracellular domain fragment (TrkB-ICD), TrkB-ICD levels were evaluated. However, no differences were observed in TrkB-ICD levels between CTL and HCD groups (CTL: 100.0 ± 11.11 %, HCD: 102.3 ± 7.341 %, *p* ≥ 0.05, *n* = 7, Figure 2f,h).

Moreover, given the significant interplay between TrkB-mediated signaling and adult hippocampal neurogenesis, an exploratory study was performed to evaluate the number of neuroblasts and immature neurons in the DG. This quantification aimed at determining if the observed changes in cognitive function and synaptic plasticity might be associated with changes in the formation of newborn adult neurons. HCD and CTL aged rats showed an identical number of DCX^+^ newborn neurons per volume (CTL: 0.133 ± 0.0586 mm^−3^, HCD: 0.159 ± 0.0513 mm^−3^, *p* ≥ 0.05, *n* = 3, Figure 2i,k). Similarly, the DG volume was not significantly different between the two experimental groups (CTL: 1307 ± 121.0 mm^3^, HCD: 2004 ± 134.5 mm^3^, *p* ≥ 0.05, *n* = 3, Figure 2j,l), and no differences were detected in the average diameter of DAPI-counterstained cell nuclei (CTL: 9.14 ± 0.190 µm, HCD: 9.13 ± 0.113 µm, *p* ≥ 0.05, *n* = 3, Figure 2j,m).

## 4. Discussion

Systematic reviews and meta-analyses clearly show that in humans, obesity has a negative impact on cognition [36,37]. However, it is still unknown what the relationship between aging and obesity on cognitive impairment is, as well as its underlying mechanisms. In this study, we show that memory and synaptic plasticity are more severely impaired in aged rats exposed to an HCD for 23 months than in age-matched CTL rats exposed to a regular diet. This strongly suggests that a high caloric intake throughout life contributes to the exacerbation and/or acceleration of the aging process, as suggested by Salvestrini et al. [38].

Obesity is characterized by body fat accumulation, with severe consequences for global health and can be clinically defined by a high body mass index [39]. The high economic costs associated with this modern pandemic are impactful, with expenses of around $2 trillion each year [4]. The weight gain of the aged rats exposed to the HCD (around 23% when compared to age-matched CTL rats) was of a similar magnitude as those previously reported using non-aged adult rats exposed to similar diets [40,41,42]. These studies also detected metabolic alterations associated with obesity (e.g., increased body mass index, body fat, cholesterol, and triglyceride levels). Moreover, in our work, CTL animals presented weight values within an expected range for Wistar rats above 21 months of age [43].

In humans, hippocampal-dependent memory, such as episodic memory, becomes rapidly impaired as a consequence of an HCD, even before weight gain [4,20]. Similarly, deficits in learning and memory tasks requiring hippocampal and cortical functions have been described in models of rodents fed HCDs, with periods of exposure ranging from days to months [20]. Although most reports account for middle-aged animals [5,44], few studies have also used aged rats briefly fed a high-fat diet [45]. In line with this evidence, in our study HCD aged rats showed long-term memory deficits in the NOR test. Given the unchanged performance of the aged rats in the OF test, the interference of locomotor difficulties was unlikely. The increased anxious-related behavior denoted in the HCD aged rats might have contributed to the diminished propensity to explore the objects in the NOR test phase and thus to the memory impairment. In fact, previous studies demonstrated enhanced anxiety-like behavior as a consequence of HCDs [46,47,48,49]. However, this evidence came from adolescent and young-adult rodents only. In agreement with our results, associations between HCDs, obesity, and mood- or anxiety-related disorders such as depression, are also suggested in humans, from adolescence to adulthood and aging [26,50,51,52,53,54,55].

The long-term recognition memory impairment observed in HCD aged rats suggests hippocampal dysfunction. The relationship between aging and impaired synaptic plasticity, namely hippocampal LTP, a well-accepted synaptic correlate of learning and memory [56], has been discussed during the last decades, with reports showing reduced synaptic plasticity associated with an age-dependent memory impairment in rodents [56,57]. Although the major risk behind cognitive decline is brain aging [58], the impact upon LTP is not completely understood, with evidence pointing to a failure of induction, maintenance and even reinforcement of LTP along the aging process [56,59]. In parallel, studies have shown a link between obesity and cognitive decline, leading, in some cases, to neurodegenerative disease, such as Alzheimer’s disease [60,61,62]. In young or adult rodents, high-fat diet exposure was shown to lead to hippocampal synaptic plasticity defects [63,64]. Herein, we show that HCD aged rats had a decreased LTP magnitude when compared with age-matched rats fed a standard diet. Interestingly, using an identical protocol of LTP induction, we previously demonstrated an enhancement in the LTP of aged rats, which likely acts as a compensatory mechanism to minimize age-associated cognitive deficits [59,65]. The present finding that an HCD lead to LTP impairment in aged rats suggests that an HCD acts as a noxious stimulus to dampen the mechanism that promotes the compensatory enhancement of synaptic plasticity during aging.

Synaptic plasticity is known to be facilitated by BDNF action through the activation of TrkB-FL [58,66,67]. Age-related decreased expression of TrkB-FL has been consistently reported [68,69] and associated with cognitive deficits [59]. Our study is the first showing decreased levels of TrkB-FL in the hippocampus of HCD aged rats when compared to age matched CTLs, clearly demonstrating that an HCD exacerbates the reduction of TrkB-FL. Additionally, besides the important role of BDNF in synaptic plasticity, this neurotrophin also regulates feeding and weight, with evidence showing that a hypomorphic or loss-of-function allele of the TrkB-FL gene triggers excessive appetite, reduced energy expenditure and morbid obesity in mice [70,71,72]. Therefore, the exacerbated decrease in the levels of these receptors observed in HCD aged rats suggests a less effective receptor activation that, besides leading to exacerbated cognitive and synaptic plasticity impairments, may even enhance appetite, thus acting as a positive feedback loop towards obesity.

Under excitotoxic age-associated conditions, such as stroke and Alzheimer’s disease, the overactivation of calpains is responsible for TrkB-FL cleavage [73,74], leading to the formation of TrkB-ICD and a transmembrane inactive form of the receptor (TrkB-T’) [74,75]. However, no difference in TrkB-ICD levels was found in HCD aged rats, suggesting that decreased levels of TrkB-FL receptors are not associated with TrkB-FL cleavage.

Adult hippocampal neurogenesis is a key contributor for brain plasticity and cognitive function, in a process highly regulated by BDNF as well as other neurotrophins [76]. Several studies demonstrated that downregulated hippocampal cell proliferation and new neuron formation are a consequence of a hypercaloric intake in rodent models of obesity [46,77,78]. However, despite the changes in BDNF-related signaling found in HCD aged rats, the number of neuroblasts/immature neurons remained unchanged in the HCD aged rats when compared with age-matched CTLs, thus suggesting preserved neurogenesis [32]. Interestingly, a specific downregulation of adult neurogenesis in female mice, but not in male mice, fed a high-fat diet has been recently proposed [79]. Importantly, further experiments with a higher number of animals are required to address this question in more detail. Nonetheless, previous studies have focused on young rodents, while HCD-induced changes in adult hippocampal neurogenesis in aged models have not been previously assessed. Given that aging is known to negatively influence neurogenesis in rodents [80,81,82], it is possible that neurogenesis is already strongly inhibited in normally fed rats so that an HCD does not lead to a further decrease.

Taken together, our results show that excessive fat and sugar intake throughout life leads to memory and synaptic plasticity decline during aging, with changes in TrkB-mediated signaling likely playing an important role. Therefore, this work further discloses mechanisms involved in the accelerated age-induced cognitive decline associated with chronic high caloric intake.

## Figures and Tables

**Table 1:** Antibodies used for Western blot and immunohistochemistry.

Antigen	Host	Supplier	Catalog Number	Dilution
**Primary antibodies**				
β-Actin (C4)	Mouse	Santa Cruz Biotechnology, Dallas, TX, USA	sc-47778	1:5000
DCX (C-18)	Goat		sc-8066	1:500
GAPDH (6C5)	Mouse	Thermo Fisher Scientific, Waltham, MA, USA	AM4300	1:5000
Trk (C-14)	Rabbit	Santa Cruz Biotechnology, Dallas, TX, USA	sc-11	1:1000
**Secondary antibodies**				
Anti-Goat Alexa Fluor^®^ 488	Donkey	Thermo Fisher Scientific, Waltham, MA, USA	A-11055	1:500
Anti-Mouse IgG-HRP	Goat	Santa Cruz Biotechnology, Dallas, TX, USA	sc-2005	1:10,000
Anti-Rabbit IgG-HRP		Bio-Rad, Hercules, CA, USA	1706515	1:10,000

## Supplementary Materials

The following are available online at https://www.mdpi.com/article/10.3390/cimb43030162/s1.
