# Supplementary material for: High Caloric Diet Induces Memory Impairment and Disrupts Synaptic Plasticity in Aged Rats
Source: Curr Issues Mol Biol. 2021 Dec 18;43(3):2305–19. doi: 10.3390/cimb43030162 (PMC8929079; doi:10.3390/cimb43030162)
Supplement: Supplementary file 1 [file cimb-43-00162-s001.zip › cimb-1478985-supplementary.pdf]

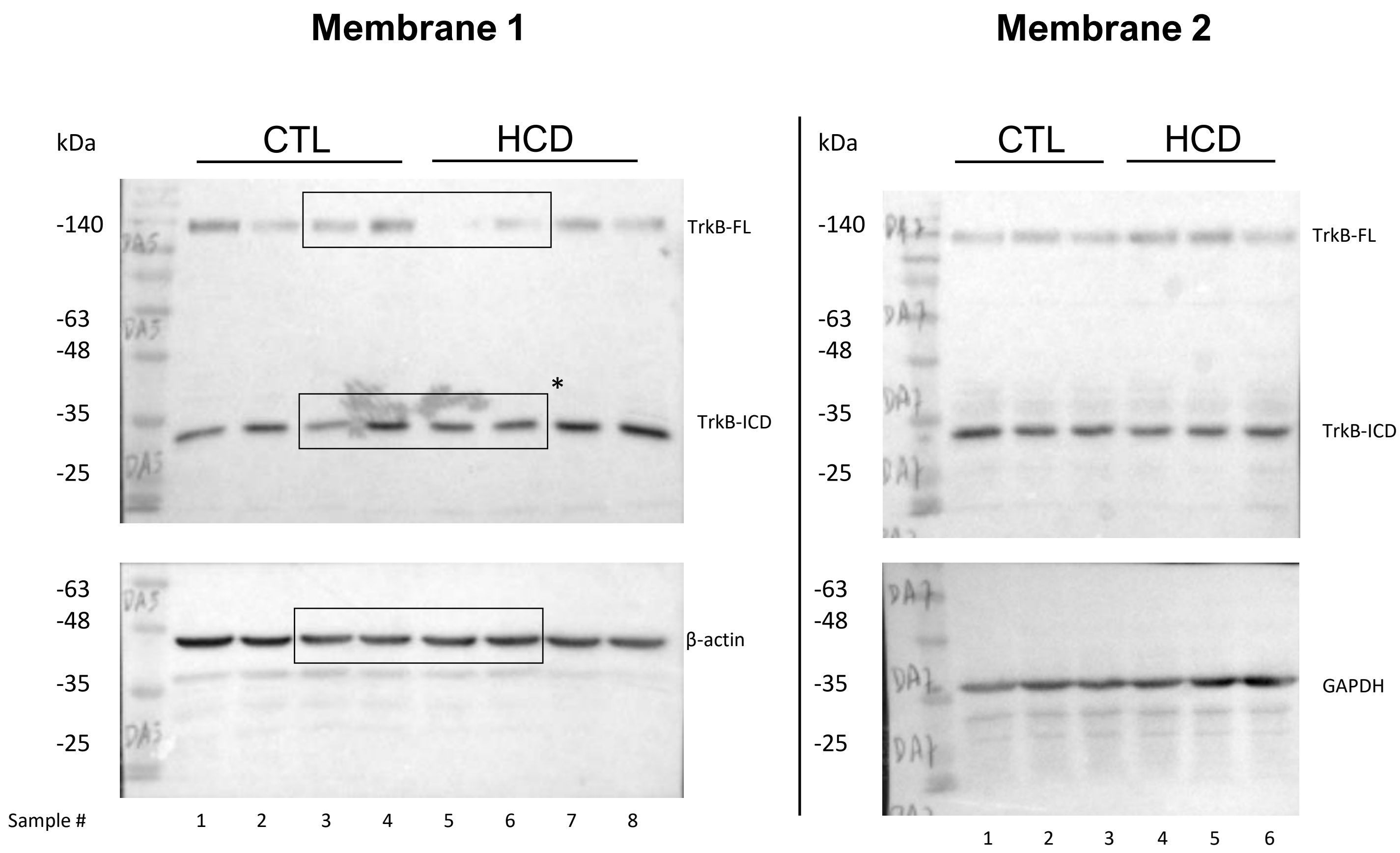

* This background was only obtained for cholorimetric picture; chemiluminescence image, the one used to quantify protein levels, did not presente this background

Representative samples

Densitometry readings

Membrane	Group	Sample #	TrkB-FL	TrkB-ICD	GAPDH	β-actin
**1**	CTL	1	382201	152455	N/A	215768
		2	190342	157916		177580
		3	247296	103043		144611
		4	362960	176731		139843
	HCD	5	37764	153038		156539
		6	156683	160553		170865
		7	233075	205306		173655
		8	202691	226244		161553
**2**	CTL	1	169372	338471	145685	N/A
		2	173969	229623	194133	
		3	159964	222629	170312	
	HCD	4	195274	205292	214540	
		5	190122	217167	270429	
		6	136109	249669	256713	
